# ZBP1 (DAI/DLM-1) promotes osteogenic differentiation while inhibiting adipogenic differentiation in mesenchymal stem cells through a positive feedback loop of Wnt/β-catenin signaling

**DOI:** 10.1038/s41413-020-0085-4

**Published:** 2020-03-05

**Authors:** Xuefeng Zhao, Liang Xie, Zhiyong Wang, Jiongke Wang, Hao Xu, Xianglong Han, Ding Bai, Peng Deng

**Affiliations:** 1 State Key Laboratory of Oral Diseases, National Clinical Research Center for Oral Diseases, Chinese Academy of Medical Sciences Research Unit of Oral Carcinogenesis and Management, West China Hospital of Stomatology, Sichuan University, Chengdu, Sichuan 610041 PR China; 2 Department of Orthodontics, West China Hospital of Stomatology, Sichuan University, Chengdu, Sichuan 610041 PR China

**Keywords:** Bone, Bone quality and biomechanics

## Abstract

The lineage specification of mesenchymal stem/stromal cells (MSCs) is tightly regulated by a wide range of factors. Recently, the versatile functions of ZBP1 (also known as DAI or DLM-1) have been reported in the blood circulation and immune systems. However, the biological function of ZBP1 during the lineage specification of MSCs is still unknown. In the present study, we found that ZBP1 was upregulated during osteogenesis but downregulated during adipogenesis in mouse bone marrow-derived MSCs (mBMSCs). ZBP1 was highly expressed in osteoblasts but expressed at a relatively low level in marrow adipocytes. Knockdown of ZBP1 inhibited alkaline phosphataseactivity, extracellular matrix mineralization, and osteogenesis-related gene expression in vitro and reduced ectopic bone formation in vivo. Knockdown of ZBP1 also promoted adipogenesis in MSCs in vitro. Conversely, the overexpression of ZBP1 increased the osteogenesis but suppressed the adipogenesis of MSCs. When the expression of ZBP1 was rescued, the osteogenic capacity of ZBP1-depleted mBMSCs was restored at both the molecular and phenotypic levels. Furthermore, we demonstrated that ZBP1, a newly identified target of Wnt/β-catenin signaling, was required for β-catenin translocation into nuclei. Collectively, our results indicate that ZBP1 is a novel regulator of bone and fat transdifferentiation via Wnt/β-catenin signaling.

## Introduction

The term Mesenchymal stem or stromal cell (MSC) refers to a group of heterogeneous multipotent stem cells that can self-renew and further differentiate into several cell types, including osteoblasts, chondrocytes, and adipocytes.^1^ They are originally derived from bone marrow and are also relatively easy to obtain from other tissue types, such as adipose tissue and umbilical cord.^2–4^ In recent years, many completed clinical trials using MSCs from bone marrow have shown the efficacy of MSC-based bone regeneration without obvious adverse effects.^5,6^

The process of MSC lineage specification is tightly regulated by many signaling pathways, transcription factors, and epigenetic mechanisms.^7–9^ For instance, the evolutionarily conserved Wnt-signaling pathway has been long known to play essential roles in MSC commitment and differentiation.^10^ Canonical Wnt signaling triggered by the binding of ligands to receptors leads to the phosphorylation of LDL receptor-related protein 5/6 coreceptors, β-catenin stabilization, and subsequent translocation into nuclei. β-Catenin with T-cell factor/lymphoid enhancer factor then promote the downstream gene expression.^11,12^ In regard to osteogenesis, Wnts promote osteoblastic differentiation, proliferation, and mineralization activity.^10,13,14^ Several osteogenesis-related transcription factors are reported to be the direct targets of Wnt/β-catenin signaling, such as Runt-related transcription factor 2 (Runx2) and *osterix* (also known as transcription factor 7, *Sp7*).^15–17^ On the other hand, activation of Wnt/β-catenin signaling also represses the adipogenic and chondrogenic differentiation of MSCs.^18,19^

Z-DNA binding protein 1 (ZBP1) is a member of the Zα family, which comprises two Zα domains at its N-terminus, and ~200 amino acids at its C-terminus.^20^ ZBP1 was initially considered to be a tumor-related protein that is dramatically upregulated in the surrounding tissue in contact with a tumor but not in the tumor tissue itself.^21^ Human *ZBP1* is highly expressed in lymphatic tissues, including the lymph nodes, bone marrow, and spleen,^22^ while the expression of mouse *Zbp1* mRNA has been reported in the lungs, liver, and spleen.^21,23^ *Zbp1* has also been found to be upregulated upon the activation of mouse peritoneal macrophages.^21^ In addition, Takaoka et al. reported that ZBP1 functions as a sensor of DNA from various sources that rapidly activates innate immune responses, during which the C terminus of ZBP1 interact with interferon (IFN) regulatory factor 3 and tank-binding kinase 1 to activate the expression of type I IFN.^24^ A recent study further reported that ZBP1 senses ribonucleoprotein complexes of influenza A viruses and becomes ubiquitinated, which is critical for inducing apoptosis, pyroptosis and necroptosis in infected tissues.^25^

However, the biological function of ZBP1 during the lineage specification of MSCs remains largely elusive. In this study, we found that ZBP1 expression was elevated during the osteogenic differentiation but downregulated during the adipogenic differentiation of MSCs. Through gain- and loss-of-function assays, we revealed that ZBP1 promoted osteogenesis but suppressed adipogenesis in MSCs. Furthermore, ZBP1 is a Wnt/β-catenin target and is required for Wnt signaling.

## Results

### ZBP1 is upregulated during the osteogenic differentiation but downregulated during the adipogenic differentiation of mBMSCs

**Fig. 1:**
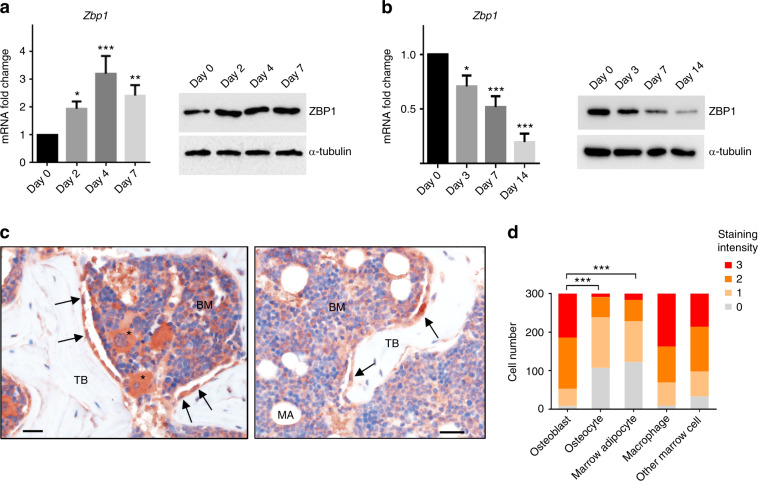
ZBP1 is upregulated during the osteogenic differentiation but downregulated during the adipogenic differentiation of mBMSCs. **a** RT-qPCR analysis and western blot analysis of ZBP1 expression during the osteogenic differentiation of mBMSCs. **b** RT-qPCR analysis and western blot analysis of ZBP1 expression during the adipogenic differentiation of mBMSCs. **c** IHC analysis of ZBP1 expression in the mouse femur. Macrophages (stars); osteoblasts on the trabecular bone surface (black arrows). *n* = 6. Bars indicate 30 μm. BM, bone marrow; TB, trabecular bone; MA, marrow adipocytes. **d** Semiquantitative quantification of ZBP1 immunostaining intensities in osteoblasts, osteocytes, marrow adipocytes, macrophages, and other marrow cells. *P < 0.05; **P < 0.01; ***P < 0.001

We first sought to examine the potential role of ZBP1 in MSC osteogenesis. As shown in Fig. 1a, ZBP1 expression was elevated upon osteogenic stimulation in mBMSCs and peaked at day 4 of induction. In contrast, it was dramatically downregulated during the adipogenesis of mBMSCs (Fig. 1b). We then evaluated the expression of ZBP1 in the mouse femurs. The immunostaining of ZBP1 showed that it was highly expressed in macrophages and osteoblasts. ZBP1 was also expressed at a relatively low level in osteocytes and marrow adipocytes (Fig. 1c, d).

### Depletion of ZBP1 suppresses the osteogenic differentiation but promotes the adipogenic differentiation of mBMSCs

**Fig. 2:**
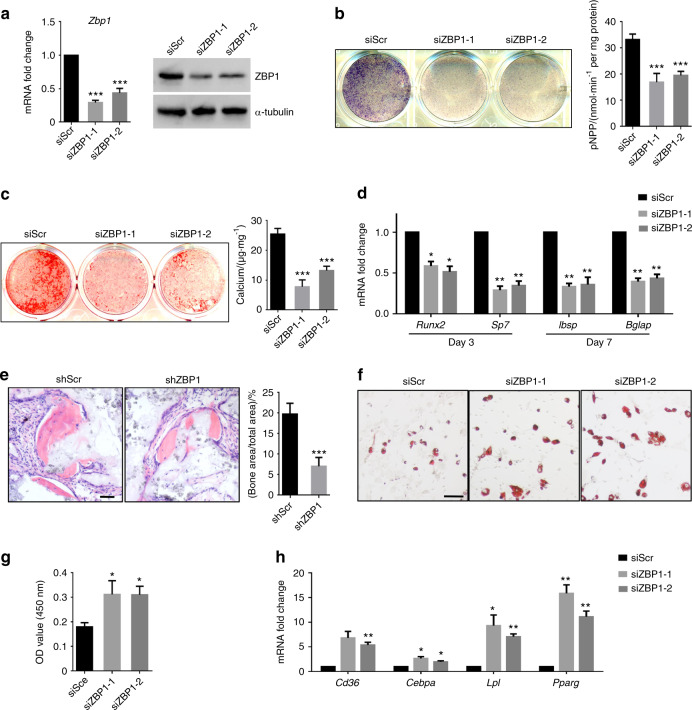
Depletion of ZBP1 suppresses the osteogenic differentiation but promotes the adipogenic differentiation of mBMSCs. **a** The siRNA-mediated depletion of ZBP1 was assessed by RT-qPCR and western blot analysis. **b** ALP activity assays of ZBP1-depleted mBMSCs and control cells after 7 days of osteogenic induction. **c** ARS staining of ZBP1-depleted mBMSCs and control cells after 2 weeks of osteogenic induction. **d** RT-qPCR analysis of osteogenesis-related genes (*Runx2*, *sp7, Ibsp*, and *Bglap*) in ZBP1-depleted mBMSCs and control cells. **e** H&E staining of transplant sections generated from ZBP1-depleted mBMSCs and control cells in HA/TCP scaffolds. Bar, 60 μm. *n* = 6. **f**, **g** Oil Red O staining of ZBP1-depleted mBMSCs and control cells after 3 weeks of adipogenic induction. Bar, 100 μm. **h** RT-qPCR analysis of adipogenic markers (*CD36, Cebpa, Lpl*, and *Pparg*) in ZBP1-depleted mBMSCs and control cells after 7 days of adipogenic induction. *P < 0.05; **P < 0.01; ***P < 0.001

Next, two specific siRNAs targeting ZBP1 was used to knock down its expression in mBMSCs (Fig. 2a). While the depletion of ZBP1 slightly inhibited the proliferation of mBMSCs (Fig. S1a), we found that ZBP1 knockdown strongly inhibited the ALP activity of mBMSCs (Fig. 2b). ECM mineralization was also reduced via the ZBP1 depletion (Fig. 2c). The expression of osteogenesis-related genes such as *Runx2, Sp7, integrin-binding sialoprotein (Ibsp)*, and *bone gamma-carboxyglutamate protein (Bglap)* was downregulated in ZBP1-depleted mBMSCs (Fig. 2d). To examine whether ZBP1 is required for in vivo bone formation, mBMSCs were infected with lentiviruses expressing ZBP1 shRNAs (shZBP1) (Fig. S1b) and subcutaneously transplanted with hydroxyapatite/tricalcium phosphate (HA/TCP) scaffolds at dorsal sites in nude mice. As shown in Fig. 2e, ectopic bone formation was inhibited in ZBP1 knowndown group in comparison with the controls. In addition, the knockdown of ZBP1 increased lipid droplet formation and the expression levels of adipogenesis-related genes in mBMSCs, including *CD36, CCAAT enhancer-binding protein alpha, lipoprotein lipase* and *peroxisome proliferator-activated receptor gamma* (Fig. 2f–h).

### Overexpression of OvereZBP1 enhances the osteogenic differentiation but inhibits the adipogenic differentiation of mBMSCs

**Fig. 3:**
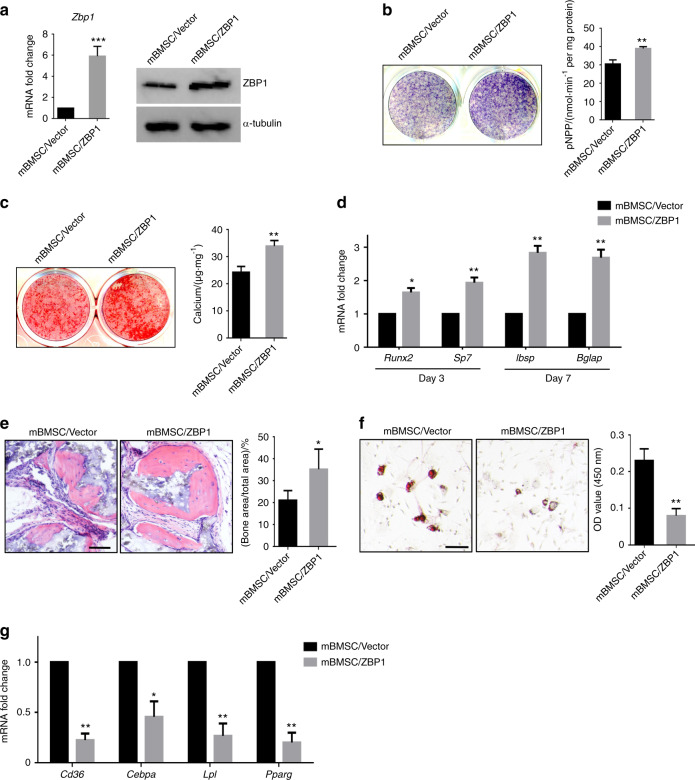
Overexpression of ZBP1 enhances the osteogenic differentiation but inhibits the adipogenic differentiation of mBMSCs. **a** The overexpression of ZBP1 was determined by RT-qPCR and western blot analysis. **b** ALP activity assays of ZBP1-overexpressing mBMSCs and control cells after 7 days of osteogenic induction. **c** ARS staining of ZBP1-overexpressing mBMSCs and control cells after 2 weeks of osteogenic stimulation. **d** RT-qPCR analysis of osteogenesis-related genes (*Runx2*, *sp7, Ibsp*, and *Bglap*) in ZBP1-overexpressing mBMSCs and control cells. **e** H&E staining of transplant sections generated from ZBP1-overexpressing mBMSCs and control cells in HA/TCP scaffolds. Bar, 60 μm. *n* = 6. **f** Oil Red O staining of ZBP1-overexpressing mBMSCs and control cells after 3 weeks of adipogenic stimulation. Bar, 100 μm. **g** RT-qPCR analysis of adipogenic markers (*CD36, Cebpa, Lpl*, and *Pparg*) in ZBP1-overexpressing mBMSCs and control cells after 7 days of adipogenic induction. *P < 0.05; **P < 0.01; ***P < 0.001

To further examine whether ZBP1 overexpression enhances the osteogenesis, mBMSCs were infected with lentiviruses expressing mouse ZBP1 (Fig. 3a). The ectopic overexpression of ZBP1 significantly increased the ALP activity of mBMSCs in response to osteogenic stimulation (Fig. 3b). Accordingly, ECM mineralization was increased in ZBP1-overexpressing mBMSCs compared with the vector control (Fig. 3c). The overexpression of ZBP1 also increased the expression levels of *Runx2*, *sp7, Ibsp*, and *Bglap* during osteogenesis in mBMSCs (Fig. 3d). Furthermore, ectopic bone formation was increased in ZBP1-overexpressing mBMSCs in comparison with control cells (Fig. 3e). On the other hand, the overexpression of ZBP1 strongly suppressed formation of lipid droplets and the expression of adipogenesis-related genes in mBMSCs (Fig. 3f, g).

### Overexpression of ZBP1 enhances the osteogenesis while inhibiting the adipogenesis of hMSCs

**Fig. 4:**
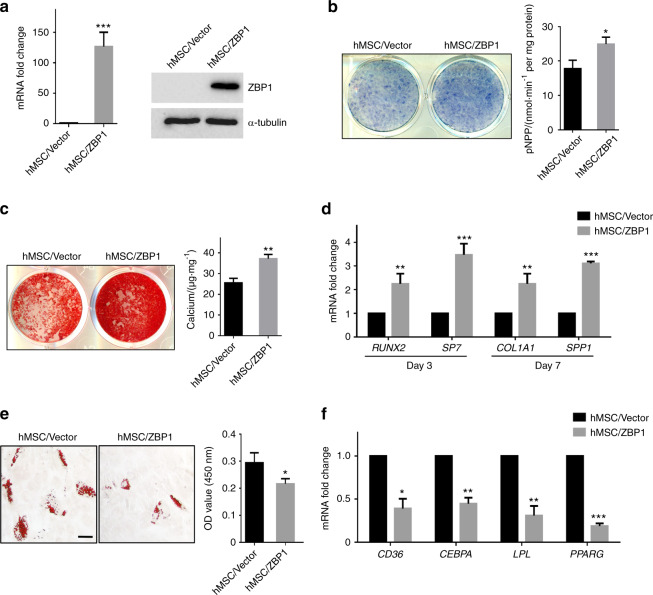
Overexpression of ZBP1 enhances the osteogenic differentiation but inhibits the adipogenic differentiation of hMSCs. **a** The overexpression of ZBP1 was determined by RT-qPCR and western blot analysis in hMSCs. **b** ALP activity assays of ZBP1-overexpressing hMSCs and control cells after 7 days of osteogenic induction. **c** ARS staining of ZBP1-overexpressing hMSCs and control cells after 3 weeks of osteogenic induction. **d** RT-qPCR analysis of osteogenic markers (*RUNX2*, *SP7, COL1A1*, and *SPP1*) in ZBP1-overexpressing hMSCs and control cells. **e** Oil Red O staining of ZBP1-overexpressing hMSCs and control cells after 4 weeks of adipogenic induction. Bar, 100 μm. **f** RT-qPCR analysis of adipogenic markers (*CD36, CEBPA, LPL*, and *PPARG*) in ZBP1-overexpressing hMSCs and control cells after 7 days of adipogenic induction. *P < 0.05; **P < 0.01; ***P < 0.001

Unlike mBMSCs, hMSCs express ZBP1 at an extremely low level. Thus, we decided to infect hMSCs with lentiviruses expressing human ZBP1 (Fig. 4a). As revealed by ALP activity assays and Alizarin Red S (ARS) staining, the overexpression of ZBP1 significantly increased the ALP activity and ECM mineralization of hMSCs (Fig. 4b, c). The expression levels of osteogenic markers, including *RUNX2*, *SP7*, *collagen type 1 alpha 1* and *secreted phosphoprotein 1* was also increased in ZBP1-overexpressing hMSCs (Fig. 4d). In contrast, we found that the overexpression of ZBP1 in hMSCs inhibited lipid droplet formation and the expression of adipogenic markers after several days of adipogenic induction (Fig. 4e, f).

### Restoring the expression of ZBP1 rescues the osteogenic potential of ZBP1-depleted mBMSCs

**Fig. 5:**
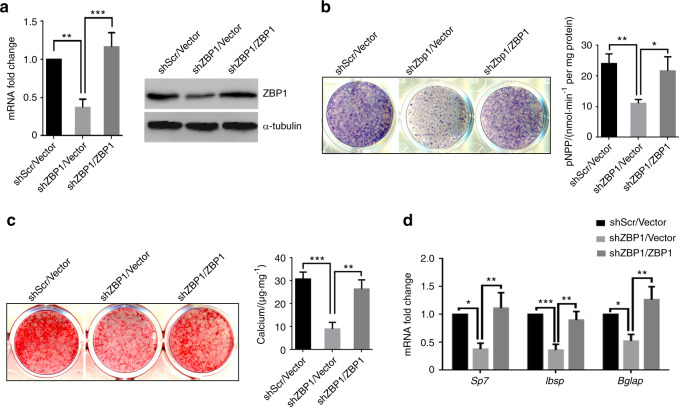
Restoring ZBP1 expression rescues the osteogenic potential of ZBP1-depleted mBMSCs. **a** RT-qPCR and western blot analysis of the restoration of ZBP1 in ZBP-depleted mBMSCs. ALP activity (**b**) and ECM mineralization (**c**) were rescued by restoring ZBP1 expression in ZBP-depleted mBMSCs. **d** The expression of osteogenic markers (*sp7, Ibsp*, and *Bglap*) was rescued by restoring ZBP1 expression in ZBP1-depleted mBMSCs. *P < 0.05; **P < 0.01; ***P < 0.001

To further confirm the specific effects of ZBP1 on the osteogenesis of MSCs, we reintroduced ZBP1 expression in mBMSCs that harbored a ZBP1 shRNA targeting the 5′ untranslated region of *Zbp1* mRNA (Fig. 5a). When mBMSC/shZBP1/ZBP1 cells were treated with osteogenic stimuli, ALP activity, and ECM mineralization were successfully rescued compared with the vector control (Fig. 5b, c). In addition, the suppressed the expression of *sp7, Ibsp*, and *Bglap* by ZBP1 depletion was also rescued when ZBP1 expression was restored (Fig. 5d). Collectively, these findings further confirmed that ZBP1 is crucial for the MSC osteogenic differentiation.

### ZBP1 interacts with β-catenin to facilitate its binding to the promoters of *Runx2* and *Sp7*

**Fig. 6:**
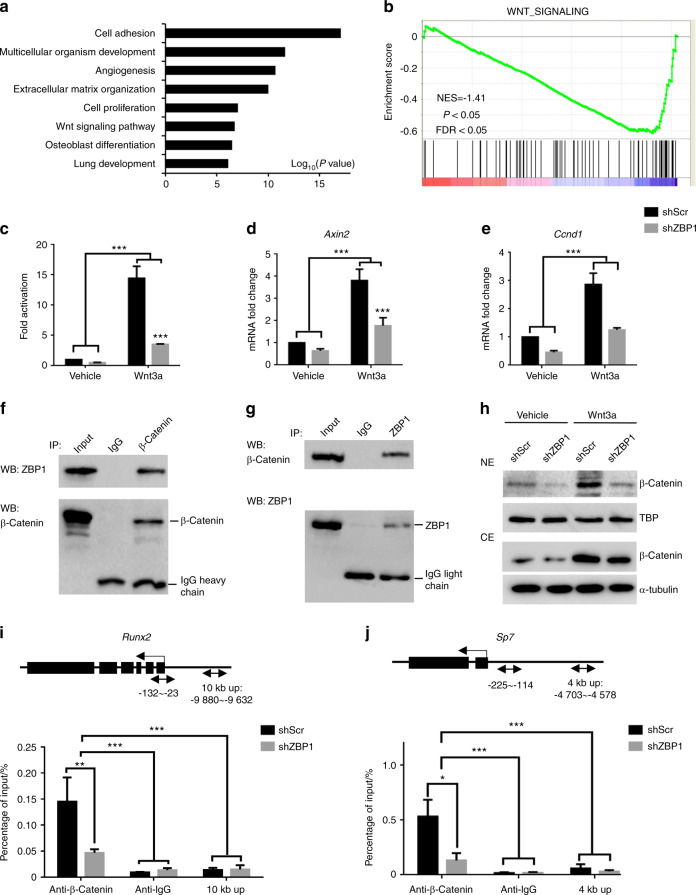
*ZBP1* is required for Wnt/β-catenin signaling. **a** GO analysis of genes downregulated (over 1.5-fold) in ZBP1-depleted mBMSCs. The top 8 ontology terms are shown. **b** GSEA of Wnt signaling-related genes in ZBP1-depleted mBMSCs compared with controls. The normalized enrichment score (NES) = −1.41, *P* < 0.05, and false discovery rate (FDR) < 0.05. **c** Topflash luciferase reporter assays of Wnt signaling activity in ZBP1-depleted mBMSCs and control cells after treatment with 100 ng·mL^−1^ Wnt3a for 24 h. RT-qPCR analysis of the expression of *Axin2* (**d**) and *Ccnd1* (**e**) in ZBP1-depleted mBMSCs and control cells after treatment with 100 ng·mL^−1^ Wnt3a for 4 h. **f**, **g** Endogenous IP reveals the interaction between ZBP1 and β-catenin in mBMSCs treated with Wnt3a (100 ng·mL^−1^) for 2 h. **h** Western blot analysis of the nuclear extract (NE) and cytoplasmic extract (CE) from ZBP1-depleted mBMSCs and control cells treated with 100 ng·mL^−1^ Wnt3a for 2 h. ChIP assay of the occupancy of β-catenin at the promoters of *Runx2* (**i**) and *Sp7* (**j**) in ZBP1-knockdown mBMSCs and control cells after treatment with 100 ng·mL^−1^ Wnt3a for 4 h. Arrows indicate primer annealing sites. *P < 0.05; **P < 0.01; ***P < 0.001

To reveal the molecular mechanisms by which ZBP1 regulates the lineage specification of mBMSCs, RNA-sequencing (RNA-seq) was performed in ZBP1-depleted mBMSCs using scrambled siRNA (siScr)-transfected mBMSCs as a control. A total of 1548 upregulated genes and 2149 downregulated genes were revealed by comparing the gene expression profiles of these cells. Gene ontology (GO) analysis revealed the downregulated genes were highly associated with ECM organization, Wnt signaling, and osteoblast differentiation (Fig. 6a, Supplementary Table S1).^26^ Gene set enrichment analysis (GSEA) further confirmed the inhibition of Wnt signaling in ZBP1-depleted mBMSCs (Fig. 6b, Supplementary Table S2).^27^

Previous studies have highlighted the essential role of ZBP1 in IFN induction by associating with TBK1 and the transcription factor IRF3.^24^ We found that ZBP1 knockdown suppressed the β-catenin-mediated transcription induced by Wnt3a treatment, as demonstrated by TOPflash luciferase reporter assays (Fig. 6c). Accordingly, the induction of *Axin2* and *Ccnd1* expression by Wnt3a treatment was also reduced in ZBP1-depleted mBMSCs (Fig. 6d, e). To examine the potential interaction between ZBP1 and β-catenin, we next performed immunoprecipitation in ZBP1-overexpressing mBMSCs. As shown in Figs. 6f, g, ZBP1 and β-catenin were coprecipitated with each other. We then obtained nuclear and cytoplasmic extracts of mBMSCs in the presence and absence of Wnt3a treatment and found that the depletion of ZBP1 significantly suppressed the nuclear translocation of β-catenin (Fig. 6h, Supplementary Fig. S2a). β-Catenin is reported to induce osteogenic differentiation through the upregulation of *Runx2* and *Sp7* expression.^17^ We confirmed that ZBP1 depletion inhibited the expression of *Runx2* and *Sp7* in mBMSCs (Supplementary Fig. S2b, c). We then designed ChIP primers that encompassed the Wnt/β-catenin response element (WRE) in the promoters of *Runx2* and *Sp7* and found that the occupancy of β-catenin at the promoters of *Runx2* and *Sp7* was significantly reduced by the depletion of ZBP1 (Figs. 6i, j).

### *Zbp1* is a direct target of Wnt/β-catenin signaling

**Fig. 7:**
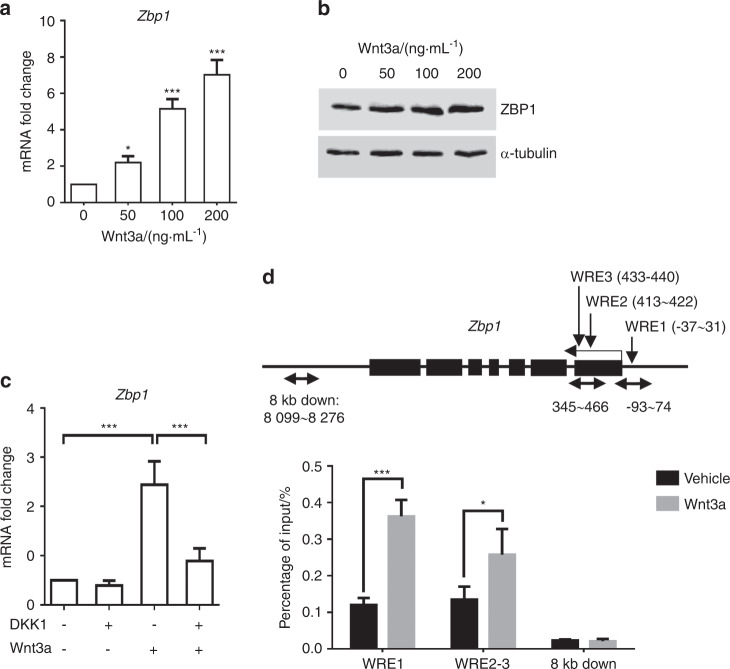
*Zbp1* is a target of Wnt/β-catenin signaling. **a** RT-qPCR analysis of *Zbp1* expression in mBMSCs treated with 100 ng·mL^−1^ Wnt3a for 4 h. **b** Western blot analysis of ZBP1 expression in mBMSCs treated with 100 ng·mL^−1^ Wnt3a for 24 h. **c** RT-qPCR analysis of *Zbp1* expression in mBMSCs that were pretreated with 100 ng·mL^−1^ DKK1 for 2 h followed by 100 ng·mL^−1^ Wnt3a treatment for 2 h. **d** ChIP assay analysis of the occupancy of β-catenin at the promoter of *Zbp1* after treatment with 100 ng·mL^−1^ Wnt3a for 4 h in mBMSCs. Arrows indicate primer annealing sites. WRE, Wnt/β-catenin response element. *P < 0.05; **P < 0.01; ***P < 0.001

Next, we showed ZBP1 expression was induced by Wnt3a treatment (Figs. 7a, b), while the induction of *Zbp1* was almost abolished by pretreatment with Dickkopf-1 (DKK1), a Wnt antagonist (Fig. 7c). Three putative WREs with the core sequence “CTTTG(A/T)(A/T)” were found within ±2 kb of the transcription start site of the mouse *Zbp1* gene.^28^ ChIP assay revealed β-catenin was recruited to the *Zbp1* promoter in Wnt3a-treated mBMSCs (Fig. 7d). Collectively, the results suggested the existence of positive feedback between ZBP1 and Wnt/β-catenin signaling that enhances osteogenic differentiation at the expense of adipogenic differentiation in MSCs.

## Discussion

Understanding MSC lineage commitment is critical for harnessing MSC differentiation into the required cell types for regenerative medicine.^29,30^ In this study, we found that ZBP1 was expressed in osteoblasts and was dramatically upregulated upon osteogenic stimulation in mBMSCs. In contrast, the expression of ZBP1 in marrow adipocytes was relatively low compared with that in osteoblasts, and ZBP1 was downregulated during the adipogenic differentiation of mBMSCs. Although ZBP1 expression in hMSCs was extremely low, the ectopic overexpression of ZBP1 strongly promoted the osteogenic but inhibited the adipogenic differentiation of hMSCs.

Our results also indicated that ZBP1 and Wnt/β-catenin signaling formed a positive feedback loop that promoted the osteogenesis while inhibiting the adipogenesis of MSCs.

Previous studies on ZBP1 have mainly focused on its structure and function in immune responses.^20,24,31,32^ To our knowledge, this is the first time that ZBP1 has been reported to be involved in the MSC lineage specification. In this study, we found that ZBP1 was also expressed in mBMSCs and their sublineages in addition to lymphatic tissues. Our results also showed that ZBP1 was essential for the osteogenic differentiation but suppressed the adipogenic differentiation of mBMSCs and hMSCs. Interestingly, while ZBP1 was highly expressed in osteoblasts, it was detected in only a small portion of osteocytes at a relatively low level. It is likely that the role of ZBP1 in regulating osteogenesis is more important in the early and middle stages compared with the late stages of osteogenesis. Unlike the pattern observed in osteogenesis, ZBP1 expression was consistently downregulated during mBMSC adipogenesis. Several previous studies have suggested a negative correlation between the osteogenesis and adipogenesis of MSCs.^8,33^ It is very likely that ZBP1 plays a key role in the early fate decision of MSCs.

We further found that *Zbp1* was a target of Wnt/β-catenin signaling and that ZBP1 was required for Wnt/β-catenin signaling. Specifically, β-catenin bound to the *Zbp1* promoter to activate its transcription, and Zbp1 in turn associated with β-catenin to facilitate its nuclear translocation and the expression of its downstream genes. As an important viral DNA sensor in innate immune responses, ZBP1 has been reported to activate IRF and nuclear factor-kappa B, promoting the production of type-I IFN.^22,34^ This study has further extended the function of ZBP1 to the facilitation of translocation of β-catenin in mBMSCs. As noted above, Wnt/β-catenin signaling may promote MSC lineage specification through multiple mechanisms. Although we focused on the binding of β-catenin to the promoters of *Runx2* and *Sp7* in this study, there might be other factors involved that maintain *Runx2* expression independent of Wnt/β-catenin signaling in untreated mBMSCs.^16,35^ On the other hand, since *Sp7* is a downstream gene of *Runx2*, these results cannot exclude the possibility that ZBP1 depletion may decrease the transcription activity of RUNX2, leading to the downregulation of *Sp7* expression.^36^ More importantly, ZBP1 may serve as a transcription factor or co-factor, since a possible association between transcriptional activity and Z-DNA formation has been suggested.^37–39^

There are several limitations of this study. For example, it has been reported that more than 2000 different transcripts might be generated from the human *ZBP1* gene by alternative splicing and/or the use of distinct 5′ and 3′ ends.^22^ Two variants are expressed from the mouse *Zbp1* gene. The siRNAs used in this study targeted both variants. However, whether different mRNAs produced by the mouse *Zbp1* gene are also involved in osteogenesis still requires further investigation. In addition, the biological function of Z-DNA is still elusive to us. Interestingly, double-stranded RNA adenosine deaminase 1, another member of the Z-DNA-binding protein family, may act as a cis-element in regulating gene expression in yeast.^40^ We also noticed that ZBP1 was expressed in both nuclei and the cytoplasm. However, additional efforts will be required to explore whether ZBP1 may directly bind to Z-DNA in promoter regions and function as a transcription factor or coactivator.

In conclusion, our findings revealed a positive feedback loop involving ZBP1 and Wnt/β-catenin signaling that promoted the osteogenesis but inhibited the adipogenesis of MSCs, indicating that ZBP1 might be a key regulator of transdifferentiation between bone and fat.

## Materials and methods

### MSC isolation and differentiation

Primary mBMSCs were from the femoral bone marrow of wild-type C57BL/6J mice at 3 months of age as previously described.^41^ The isolated cells were maintained in Dulbecco’s modified Eagle’s medium, containing 15% heat-inactivated fetal bovine serum, 100 U·mL^−1^ of penicillin and 100 µg·mL^−1^ of streptomycin sulfate for 3 days (all from Invitrogen). The nonadherent hematopoietic cells were then removed by vigorous washing with phosphate-buffered saline (PBS) three times. The growing colonies were collected by trypsinization after 2 weeks for further passaging and differentiation. The in vivo and in vitro animal procedures were approved by the Committee for the Care and Use of Laboratory Animals of the State Key Laboratory of Oral Diseases, West China Hospital of Stomatology, Sichuan University.

hMSCs were purchased from the ATCC and were maintained in Dulbecco’s modified Eagle’s medium supplemented with 15% FBS plus 100 U·mL^−1^ penicillin and 100 µg·mL^−1^ streptomycin sulfate (all from Invitrogen).

### Induction of osteogenesis and characterization of osteogenic phenotypes

A total of 1 × 10^5^ cells per well were seeded into 12-well plates and cultured in osteogenic induction medium, which is consisted of 90% α-MEM (Invitrogen) supplemented with 10% FBS (Invitrogen), 100 μmol·L^−1^ vitamin C (Sigma-Aldrich), 10 nmol·L^−1^ dexamethasone (Sigma-Aldrich), and 10 mmol·L^−1^ β-glycerophosphate (Sigma-Aldrich).

After several days of osteogenic stimulation, ALP staining, ALP activity assay, and ARS staining was carried out as previously described.^29^

### Induction of adipogenesis and characterization of adipogenic phenotypes

At 95%–100% cell confluence, the cells were treated with adipogenic induction medium, and Oil Red O staining was carried out after 2–4 weeks of stimulation to measure the formation of lipid droplets in the cells using a kit from Diagnostic Biosystems.^42^

### Immunohistochemistry (IHC)

Femurs from 3-month-old mice were collected and fixed with 10% neutral buffered formalin (Fisher) for 48 h. After decalcification with a 10% ethylenediaminetetraacetic acid solution (pH = 7.4) for 2 weeks, paraffin sections were obtained, and the specimens were then dewaxed with xylene twice and rehydrated through a gradient from ethanol to PBS. Immunostaining was performed using an IHC kit (Gene Tech) according to the manufacturers’ instructions. A rabbit polyclonal anti-ZBP1 antibody (Invitrogen) was used to detect the expression of ZBP1 in bone marrow and trabecular bones. The semiquantitative quantification of immunostaining intensities (0, negative; 1, weak staining; 2, moderate staining; 3, intense staining) was independently carried out by two pathologists as previously reported.^43,44^

### 3-(4,5-dimethylthiazol-2-yl)-2,5-diphenyltetrazolium bromide (MTT) assay

A total of 3 × 10^3^ cells were seeded into each well of 96-well plates in at least triplicate and cultured until the indicated time points posttransfection with siRNAs. Overall, 10 µL of MTT (Sigma-Aldrich) at a concentration of 5 mg·mL^−1^ was added, followed by incubation for additional 3 h. The medium was then replaced with dimethyl sulfoxide, and the optical absorbance was read at 570 nm.

### Reverse transcription-quantitative PCR (RT-qPCR)

RNA was isolated using the TRIzol reagent (Invitrogen). For each sample, complementary DNA was generated from 1 to 2 µg of total RNA using PrimeScript RT Reagent Kit (TAKARA BIO). Please refer to Supplementary Table S3 for the primers used for RT-qPCR.

### Western blotting

The cells were rinsed with cold PBS and lysed in RIPA buffer (Boster Biological Technology, Wuhan, China) for 30 min with constant agitation. A 10–40 μg aliquot of the supernatant from each sample mixed with loading dye was boiled at 95 °C for 4 min and then separated on sodium dodecyl sulfate-polyacrylamide gels at 80–120 V. Western blotting was carried out as previously described.^42^ The primary antibodies used included a rabbit anti-ZBP1 antibody (Invitrogen), a mouse anti-β-catenin antibody (BD Biosciences), a mouse anti-TATA-binding protein TBP antibody (Abcam), and a mouse anti-α-tubulin antibody (Sigma-Aldrich).

### Transfection and viral infection

All the siRNAs used in this study were obtained from Origene Technologies, including a siScr and two siRNAs targeting mouse ZBP1 (siZBP1–1, siZBP1–2). More specifically, siZBP1–1 targeted the open reading frame of *Zbp1* mRNA, and its sequence was “GGGAAUGACGACAGCCAAA”. siZBP1–2 targeted the 5′ untranslated region (UTR) of *Zbp1* mRNA, and its sequence was “GGGUAUUUGUUUCCGGGAU”. Transfection with the siRNAs was conducted by using Lipofectamine RNAiMAX reagent (Invitrogen). For the long-term knockdown of ZBP1 and rescue experiments, lentiviruses expressing ZBP1 shRNA (shZBP1) were generated by Fulengen Inc. (Guangzhou, China). For the ectopic overexpression of ZBP1, lentivirus particles expressing mouse ZBP1 and human ZBP1 were obtained from OriGene Technologies. mBMSCs and hMSCs were infected and then selected with 1 μg·mL^−1^ puromycin for 72 h.

### Luciferase assay

mBMSCs were seeded into 12-well plates and infected with lentiviruses carrying TOPflash luciferase reporters. After treatment with Wnt3a at 100 ng·mL^−1^ for 1 day, luciferase activity was assessed using Luc-Screen kits (Tropix) as previously described.^45^

### Immunoprecipitation (IP) assay

Protein was isolated from ZBP1-overexpressing mBMSCs. Aliquots of 50–100 μg of protein were incubated with 1 µg of antibodies overnight at 4 °C with gentle rotation. The antigen-antibody complex was then precipitated with Dynabeads Protein A/G (Thermo). The beads carrying the antigen-antibody complexes were washed at least three times to remove nonspecific binding and were then subjected to western blotting. The antibodies used for the IP experiments were as follows: mouse monoclonal anti-β-catenin (BD Biosciences), polyclonal anti-mouse normal IgG (Millipore), rabbit polyclonal anti-ZBP1 (LifeSpan BioSciences), and polyclonal anti-rabbit normal IgG (Cell Signaling Technology).

### ChIP assay

ChIP experiments were carried out according to the manufacturer’s protocol (Zymo Research, USA). More specifically, cells were collected and crosslinked with 1% formaldehyde for 15 min with gentle rotation. Nuclei were then extracted using Nuclei Prep buffer. The isolated nuclei were sheared via 3–5 cycles of sonication. After centrifugation, the supernatant containing the sheared chromatin was incubated with the antibodies overnight at 4 °C under rotation. ZymoMag Protein A4 beads were added to the reaction. Finally, the precipitated DNA was eluted from the beads and purified for quantification by qPCR. The amount of ChIP-ed DNA was normalized to the input DNA of each sample. The following antibodies were used: mouse monoclonal anti-β-catenin (BD Biosciences, 1:200) and anti-mouse IgG (Millipore, 1:500). The primers used in ChIP assay are also shown in Supplementary Table S3.

### Transplantation in nude mice

mBMSCs (1 × 10^6^) expressing shZBP1 and shScr were incubated with 40 mg of HA/TCP scaffolds at 37 °C for 6 h and then subcutaneously transplanted into female nude mice (*n* = 6).^46^ The transplants were collected at 6 weeks post transplantation. At least three fields of each H&E-stained sample were randomly chosen (Olympus), and the area of bone tissue versus the total area was measured using SPOT 4.0 software.

### RNA-seq

RNA was collected from duplicated mBMSCs transfected with siScrs and siRNAs targeting ZBP1 at 72 h posttransfection. RNA-seq libraries were prepared using the Illumina TruSeq RNA Sample Preparation Kit. All the sequenced reads were mapped to the NCBI build 10 mm genome using Tophat v2.0.12, and fragments per kilobase of transcript per million mapped reads (FPKM) expression values were extracted using HTSeq. GSEA and GO analysis were performed to identify the genes that were downregulated by 1.5-fold in ZBP1-depleted mBMSCs.^26,27^ The accession number is NCBI GEO: GSE117334.

### Statistical analysis

Data are presented as the mean ± SD. *P < 0.05; **P < 0.01; ***P < 0.001. Student’s *t* test was used for single comparisons; one-way analysis of variance (ANOVA) and two-way ANOVA was performed for multiple comparisons. The chi-square test was performed to examine the significant differences between the immunostaining intensities of different cell types in the mouse femur.

## Supplementary information

Supplementary materials

Figure S1

Figure S2

## Data Availability

All the data is included in the published files.

## References

[1] Nombela-Arrieta C, Ritz J, Silberstein LE (2011). The elusive nature and function of mesenchymal stem cells. Mol. Cell Biol..

[2] Koc ON (1999). Bone marrow-derived mesenchymal stem cells remain host-derived despite successful hematopoietic engraftment after allogeneic transplantation in patients with lysosomal and peroxisomal storage diseases. Exp. Hematol..

[3] Lee OK (2004). Isolation of multipotent mesenchymal stem cells from umbilical cord blood. Blood.

[4] Lee RH (2004). Characterization and expression analysis of mesenchymal stem cells from human bone marrow and adipose tissue. Cell Physiol. Biochem..

[5] Kim N, Cho SG (2013). Clinical applications of mesenchymal stem cells. Korean J. Intern. Med..

[6] Wang S, Qu X, Zhao RC (2012). Clinical applications of mesenchymal stem cells. J. Hematol. Oncol..

[7] Neve A, Corrado A, Cantatore FP (2011). Osteoblast physiology in normal and pathological conditions. Cell Tissue Res..

[8] Deng P, Chen Q-M, Hong C, Wang C-Y (2015). Histone methyltransferases and demethylases: regulators in balancing osteogenic and adipogenic differentiation of mesenchymal stem cells. Int. J. Oral. Sci..

[9] Liu W (2016). GDF11 decreases bone mass by stimulating osteoclastogenesis and inhibiting osteoblast differentiation. Nat. Commun..

[10] Krishnan V, Bryant HU, MacDougald OA (2006). Regulation of bone mass by Wnt signaling. J. Clin. Investig..

[11] Hay E (2005). Interaction between LRP5 and Frat1 mediates the activation of the wnt canonical pathway. J. Biol. Chem..

[12] Levy L., Wei Y., Labalette C., Wu Y., Renard C.-A., Buendia M. A., Neuveut C. (2004). Acetylation of  -Catenin by p300 Regulates  -Catenin-Tcf4 Interaction. Molecular and Cellular Biology.

[13] Bennett CN (2005). Regulation of osteoblastogenesis and bone mass by Wnt10b. Proc. Natl Acad. Sci. USA.

[14] Jackson A (2005). Gene array analysis of Wnt-regulated genes in C3H10T1/2 cells. Bone.

[15] Gaur T (2005). Canonical WNT signaling promotes osteogenesis by directly stimulating Runx2 gene expression. J. Biol. Chem..

[16] Rodríguez-Carballo E (2011). Conserved regulatory motifs in osteogenic gene promoters integrate cooperative effects of canonical Wnt and BMP pathways. J. Bone Miner. Res..

[17] Liu B, Wu S, Han L, Zhang C (2015). beta-catenin signaling induces the osteoblastogenic differentiation of human pre-osteoblastic and bone marrow stromal cells mainly through the upregulation of osterix expression. Int. J. Mol. Med..

[18] Ross SE (2000). Inhibition of adipogenesis by Wnt signaling. Science.

[19] Day TF, Guo X, Garrett-Beal L, Yang Y (2005). Wnt/beta-catenin signaling in mesenchymal progenitors controls osteoblast and chondrocyte differentiation during vertebrate skeletogenesis. Dev. Cell.

[20] Schwartz T, Behlke J, Lowenhaupt K, Heinemann U, Rich A (2001). Structure of the DLM-1–Z-DNA complex reveals a conserved family of Z-DNA-binding proteins. Nat. Struct. Biol..

[21] Fu Y (1999). Cloning of DLM-1, a novel gene that is up-regulated in activated macrophages, using RNA differential display. Gene.

[22] Rothenburg S, Schwartz T, Koch-Nolte F, Haag F (2002). Complex regulation of the human gene for the Z-DNA binding protein DLM-1. Nucleic Acids Res..

[23] Yue F (2014). A comparative encyclopedia of DNA elements in the mouse genome. Nature.

[24] Takaoka A (2007). DAI (DLM-1/ZBP1) is a cytosolic DNA sensor and an activator of innate immune response. Nature.

[25] Kesavardhana S (2017). ZBP1/DAI ubiquitination and sensing of influenza vRNPs activate programmed cell death. J. Exp. Med.

[26] Huang DW, Sherman BT, Lempicki RA (2008). Systematic and integrative analysis of large gene lists using DAVID bioinformatics resources. Nat. Protoc..

[27] Subramanian A (2005). Gene set enrichment analysis: a knowledge-based approach for interpreting genome-wide expression profiles. Proc. Natl Acad. Sci. USA.

[28] Mathelier A (2016). JASPAR 2016: a major expansion and update of the open-access database of transcription factor binding profiles. Nucleic Acids Res..

[29] Zhao X (2016). Cysteine dioxygenase type 1 inhibits osteogenesis by regulating Wnt signaling in primary mouse bone marrow stromal cells. Sci. Rep..

[30] Bianco P (2013). The meaning, the sense and the significance: translating the science of mesenchymal stem cells into medicine. Nat. Med..

[31] DeFilippis VR, Alvarado D, Sali T, Rothenburg S, Früh K (2010). Human cytomegalovirus induces the interferon response via the DNA sensor ZBP1. J. Virol..

[32] Kuriakose T, Kanneganti T-D (2018). ZBP1: innate sensor regulating cell death and inflammation. Trends Immunol..

[33] Beresford JN, Bennett JH, Devlin C, Leboy PS, Owen ME (1992). Evidence for an inverse relationship between the differentiation of adipocytic and osteogenic cells in rat marrow stromal cell cultures. J. Cell Sci..

[34] Rebsamen M (2009). DAI/ZBP1 recruits RIP1 and RIP3 through RIP homotypic interaction motifs to activate NF-kappaB. EMBO Rep..

[35] Jonason JH, Xiao G, Zhang M, Xing L, Chen D (2009). Post-translational regulation of Runx2 in bone and cartilage. J. Dent. Res..

[36] Nakashima K (2002). The novel zinc finger-containing transcription factor osterix is required for osteoblast differentiation and bone formation. Cell.

[37] Wölfl S, Wittig B, Dorbic T, Rich A (1997). Identification of processes that influence negative supercoiling in the human c-myc gene. Biochim. Biophys. Acta, Gene Struct. Expr..

[38] Wittig B, Wölfl S, Dorbic T, Vahrson W, Rich A (1992). Transcription of human c-myc in permeabilized nuclei is associated with formation of Z-DNA in three discrete regions of the gene. EMBO J..

[39] Wölfl S, Martinez C, Rich A, Majzoub JA (1996). Transcription of the human corticotropin-releasing hormone gene in NPLC cells is correlated with Z-DNA formation. Proc. Nat. Acad. Sci. USA.

[40] Oh DB, Kim YG, Rich A (2002). Z-DNA-binding proteins can act as potent effectors of gene expression in vivo. Proc. Nat. Acad. Sci. USA.

[41] Soleimani M, Nadri S (2009). A protocol for isolation and culture of mesenchymal stem cells from mouse bone marrow. Nat. Protoc..

[42] Deng P (2015). Cysteine dioxygenase type 1 promotes adipogenesis via interaction with peroxisome proliferator-activated receptor gamma. Biochem. Biophys. Res. Commun..

[43] Detre S, Saclani Jotti G, Dowsett M (1995). A “quickscore” method for immunohistochemical semiquantitation: validation for oestrogen receptor in breast carcinomas. J. Clin. Pathol..

[44] Deng P (2018). AFF4 promotes tumorigenesis and tumor-initiation capacity of head and neck squamous cell carcinoma cells by regulating SOX2. Carcinogenesis.

[45] Li J (2017). KDM3 epigenetically controls tumorigenic potentials of human colorectal cancer stem cells through Wnt/β-catenin signalling. Nat. Commun..

[46] Deng P, Zhou C, Alvarez R, Hong C, Wang CY (2016). Inhibition of IKK/NF-kappaB signaling enhances differentiation of mesenchymal stromal cells from human embryonic stem cells. Stem Cell Rep..

