# Supplementary material for: ZBP1 (DAI/DLM-1) promotes osteogenic differentiation while inhibiting adipogenic differentiation in mesenchymal stem cells through a positive feedback loop of Wnt/β-catenin signaling
Source: Bone Res. 2020 Mar 5;8:12. doi: 10.1038/s41413-020-0085-4 (PMC7058036; doi:10.1038/s41413-020-0085-4)
Supplement: Supplementary file 1 — Supplementary materials [file 41413_2020_85_MOESM1_ESM.docx]

**ZBP1 (DAI/DLM-1) promotes osteogenic but inhibits adipogenic differentiation of mesenchymal stem cells through a positive feedback loop of Wnt/β-Catenin signaling**

Running Title: *ZBP1 regulates MSC osteo- and adipo-genesis*

Xuefeng Zhao^1,2^, Liang Xie^1^, Zhiyong Wang^1^, Jiongke Wang^1^, Hao Xu^1^, Xianglong Han^1^, Ding Bai^1,2*^, Peng Deng^1*^

List of supplementary information:

1. Supplementary Table S1, related Fig. 6a
2. Supplementary Table S2, related Fig. 6b
3. Supplementary Table S3, related to all qPCR experiments.
4. Fig. S1, related to Fig. 1
5. Fig. S2, related to Fig. 6

Supplementary Table S1. Down-regulated Wnt signaling-related genes in ZBP1-depleted mBMSCs, compared to scrambled siRNA-transfected mBMSCs. Related to Fig. 6a.

| GENE SYMBOL | Gene Description | Average FPKM | | Fold Change |
| --- | --- | --- | --- | --- |
|  |  | scramble | siZBP1 |  |
| *Amotl1* | angiomotin-like 1 | 8.642 | 3.356 | 0.388 |
| *Apcdd1* | adenomatosis polyposis coli down-regulated 1 | 3.532 | 2.081 | 0.589 |
| *Axin2* | axin 2 | 1.704 | 1.129 | 0.663 |
| *Ccdc88c* | coiled-coil domain containing 88C | 0.222 | 0.057 | 0.256 |
| *Ccnd1* | cyclin D1 | 107.502 | 50.882 | 0.473 |
| *Ccne1* | cyclin E1 | 6.723 | 4.431 | 0.659 |
| *Csnk1g1* | casein kinase 1, gamma 1 | 9.823 | 6.182 | 0.629 |
| *Ddit3* | DNA-damage inducible transcript 3 | 69.526 | 33.611 | 0.483 |
| *Dixdc1* | DIX domain containing 1 | 1.353 | 0.825 | 0.610 |
| *Dkk3* | dickkopf WNT signaling pathway inhibitor 3 | 191.819 | 117.353 | 0.612 |
| *Draxin* | dorsal inhibitory axon guidance protein | 0.220 | 0.067 | 0.305 |
| *Fermt2* | fermitin family member 2 | 57.342 | 33.433 | 0.583 |
| *Frzb* | frizzled-related protein | 0.618 | 0.348 | 0.563 |
| *Fzd5* | frizzled class receptor 5 | 15.590 | 8.708 | 0.559 |
| *Fzd7* | frizzled class receptor 7 | 11.724 | 6.732 | 0.574 |
| *Fzd8* | frizzled class receptor 8 | 7.371 | 4.066 | 0.552 |
| *Fzd9* | frizzled class receptor 9 | 0.161 | 0.047 | 0.295 |
| *Kremen1* | kringle containing transmembrane protein 1 | 25.529 | 12.785 | 0.501 |
| *Lrp4* | low density lipoprotein receptor-related protein 4 | 12.909 | 4.075 | 0.316 |
| *Myc* | myelocytomatosis oncogene | 21.449 | 9.713 | 0.453 |
| *Nkd1* | naked cuticle 1 homolog | 0.699 | 0.335 | 0.480 |
| *Notum* | notum pectinacetylesterase homolog | 0.222 | 0.026 | 0.116 |
| *Nxn* | nucleoredoxin | 19.975 | 12.282 | 0.615 |
| *Peg12* | paternally expressed 12 | 1.603 | 0.889 | 0.555 |
| *Prkaa1* | protein kinase, AMP-activated, alpha 1 catalytic subunit | 22.616 | 12.359 | 0.546 |
| *Pygo1* | pygopus 1 | 2.033 | 1.158 | 0.570 |
| *Ror2* | receptor tyrosine kinase-like orphan receptor 2 | 4.347 | 2.485 | 0.572 |
| *Rspo2* | R-spondin 2 | 40.471 | 12.995 | 0.321 |
| *Rspo3* | R-spondin 3 | 0.869 | 0.314 | 0.361 |
| *Ryk* | receptor-like tyrosine kinase | 34.812 | 15.636 | 0.449 |
| *Sfrp1* | secreted frizzled-related protein 1 | 7.552 | 2.165 | 0.287 |
| *Sfrp2* | secreted frizzled-related protein 2 | 18.120 | 1.718 | 0.095 |
| *Sfrp4* | secreted frizzled-related protein 4 | 6.318 | 3.236 | 0.512 |
| *Sox17* | SRY (sex determining region Y)-box 17 | 0.099 | 0.024 | 0.245 |
| *Tcf7* | transcription factor 7, T cell specific | 4.863 | 1.473 | 0.303 |
| *Tgfb1i1* | transforming growth factor beta 1 induced transcript 1 | 19.588 | 6.748 | 0.344 |
| *Tle2* | transducin-like enhancer of split 2 | 2.907 | 1.885 | 0.649 |
| *Trabd2b* | TraB domain containing 2B | 0.699 | 0.165 | 0.236 |
| *Wif1* | Wnt inhibitory factor 1 | 5.235 | 0.477 | 0.091 |
| *Wisp1* | WNT1 inducible signaling pathway protein 1 | 82.611 | 43.924 | 0.532 |
| *Wnt10b* | wingless-type MMTV integration site family, member 10B | 6.331 | 1.446 | 0.228 |
| *Wnt11* | wingless-type MMTV integration site family, member 11 | 0.686 | 0.222 | 0.323 |
| *Wnt16* | wingless-type MMTV integration site family, member 16 | 1.631 | 0.640 | 0.392 |
| *Wnt4* | wingless-type MMTV integration site family, member 4 | 2.045 | 0.508 | 0.248 |

Supplementary Table S2. Expression of genes in Wnt signaling gene set used for GSEA analysis. Related to Fig. 6b.

| Gene Symbol | Gene Description | Average FPKM | | Fold Change |
| --- | --- | --- | --- | --- |
|  |  | scramble | siZBP1 |  |
| *Actb* | actin, beta | 1982.200 | 1929.015 | 0.973 |
| *Aes* | amino-terminal enhancer of split | 145.897 | 155.418 | 1.065 |
| *Apc* | adenomatous polyposis coli | 13.277 | 10.519 | 0.792 |
| *Axin1* | axin 1 | 14.287 | 14.157 | 0.991 |
| *B2m* | beta-2-microglobulin | 4666.865 | 4462.040 | 0.956 |
| *Bcl9* | B-cell CLL/lymphoma 9 | 5.411 | 3.835 | 0.709 |
| *Btrc* | beta-transducin repeat containing | 3.218 | 3.695 | 1.148 |
| *Ccnd1* | cyclin D1 | 107.502 | 50.882 | 0.473 |
| *Ccnd2* | cyclin D2 | 122.419 | 93.191 | 0.761 |
| *Ccnd3* | cyclin D3 | 29.616 | 33.265 | 1.123 |
| *Csnk1a1* | casein kinase 1, alpha 1 | 114.665 | 118.238 | 1.031 |
| *Csnk1d* | casein kinase 1, delta | 41.437 | 45.030 | 1.087 |
| *Csnk1g1* | casein kinase 1, gamma 1 | 9.823 | 6.182 | 0.629 |
| *Csnk2a1* | casein kinase 2, alpha 1 polypeptide | 38.744 | 34.738 | 0.897 |
| *Ctbp1* | C-terminal binding protein 1 | 66.949 | 61.520 | 0.919 |
| *Ctbp2* | C-terminal binding protein 2 | 22.936 | 21.723 | 0.947 |
| *Ctnnb1* | catenin beta 1 | 227.034 | 248.628 | 1.095 |
| *Ctnnbip1* | catenin, beta interacting protein 1 | 6.729 | 6.700 | 0.996 |
| *Cxxc4* | CXXC finger protein 4 | 0.000 | 0.000 | N/A |
| *Daam1* | dishevelled associated activator of morphogenesis 1 | 7.759 | 8.247 | 1.063 |
| *Dixdc1* | DIX domain containing 1 | 1.353 | 0.825 | 0.610 |
| *Dkk1* | dickkopf homolog 1 (Xenopus laevis) | 0.193 | 0.149 | 0.772 |
| *Dvl1* | dishevelled, dsh homolog 1 (Drosophila) | 12.588 | 12.023 | 0.955 |
| *Dvl2* | dishevelled, dsh homolog 2 (Drosophila) | 8.515 | 7.976 | 0.937 |
| *EP300* | E1A binding protein p300 | 27.029 | 27.575 | 1.020 |
| *Fbxw11* | F-box and WD repeat domain containing 11 | 30.691 | 33.604 | 1.095 |
| *Fbxw2* | F-box and WD repeat domain containing 2 | 23.980 | 21.868 | 0.912 |
| *Fbxw4* | F-box and WD repeat domain containing 4 | 9.550 | 9.389 | 0.983 |
| *Fgf4* | fibroblast growth factor 4 | 0.012 | 0.000 | 0.000 |
| *Fosl1* | FOS-like antigen 1 | 1.793 | 0.810 | 0.452 |
| *Foxn1* | forkhead box N1 | 0.075 | 0.000 | 0.000 |
| *Frat1* | frequently rearranged in advanced T cell lymphomas | 0.942 | 1.494 | 1.587 |
| *Frzb* | frizzled-related protein | 0.618 | 0.348 | 0.563 |
| *Fshb* | follicle stimulating hormone subunit beta | 0.000 | 0.000 | N/A |
| *Fzd1* | frizzled family receptor 1 | 15.938 | 12.046 | 0.756 |
| *Fzd2* | frizzled family receptor 2 | 9.865 | 10.545 | 1.069 |
| *Fzd3* | frizzled family receptor 3 | 1.392 | 1.296 | 0.931 |
| *Fzd4* | frizzled family receptor 4 | 0.656 | 0.651 | 0.992 |
| *Fzd5* | frizzled family receptor 5 | 15.590 | 8.708 | 0.559 |
| *Fzd6* | frizzled family receptor 6 | 1.258 | 2.944 | 2.340 |
| *Fzd7* | frizzled family receptor 7 | 11.724 | 6.732 | 0.574 |
| *Fzd8* | frizzled family receptor 8 | 7.371 | 4.066 | 0.552 |
| *Gapdh* | glyceraldehyde-3-phosphate dehydrogenase | 552.728 | 645.355 | 1.168 |
| *Gsk3a* | glycogen synthase kinase 3 alpha | 28.541 | 30.155 | 1.057 |
| *Gsk3b* | glycogen synthase kinase 3 beta | 25.595 | 23.796 | 0.930 |
| *Hprt1* | hypoxanthine phosphoribosyltransferase 1 | 117.006 | 128.549 | 1.099 |
| *Jun* | jun proto-oncogene | 48.132 | 50.463 | 1.048 |
| *Kreme1* | kringle containing transmembrane protein 1 | 25.529 | 12.785 | 0.501 |
| *Lef1* | lymphoid enhancer-binding factor 1 | 0.163 | 0.306 | 1.880 |
| *Lrp5* | low density lipoprotein receptor related protein 5 | 8.789 | 8.605 | 0.979 |
| *Lrp6* | low density lipoprotein receptor related protein 6 | 16.442 | 16.239 | 0.988 |
| *Myc* | v-myc myelocytomatosis viral related oncogene | 21.449 | 9.713 | 0.453 |
| *Nkd1* | naked cuticle homolog 1 (Drosophila) | 0.699 | 0.335 | 0.480 |
| *Nlk* | nemo-like kinase | 7.475 | 7.883 | 1.055 |
| *Pitx2* | paired-like homeodomain 2 | 0.000 | 0.015 | N/A |
| *Porcn* | porcupine homolog (Drosophila) | 14.765 | 17.258 | 1.169 |
| *Ppp2ca* | protein phosphatase 2, catalytic subunit alpha | 110.526 | 114.954 | 1.040 |
| *Ppp2r1a* | protein phosphatase 2, scaffold subunit alpha | 105.687 | 113.294 | 1.072 |
| *Pygo1* | pygopus homolog 1 (Drosophila) | 2.033 | 1.158 | 0.570 |
| *Rhou* | ras homolog gene family, member U | 4.008 | 1.214 | 0.303 |
| *Rpl13a* | ribosomal protein L13a | 633.235 | 529.713 | 0.837 |
| *Senp2* | SUMO1/sentrin/SMT3 specific peptidase 2 | 21.660 | 27.458 | 1.268 |
| *Sfrp1* | secreted frizzled-related protein 1 | 7.552 | 2.165 | 0.287 |
| *Sfrp4* | secreted frizzled-related protein 4 | 6.318 | 3.236 | 0.512 |
| *Slc9a3r1* | solute carrier family 9 (sodium/hydro... | 35.691 | 39.732 | 1.113 |
| *Sox17* | SRY (sex determining region Y)-box 17 | 0.099 | 0.024 | 0.245 |
| *T* | T, brachyury homolog (mouse) | 0.022 | 0.000 | 0.000 |
| *Tcf7* | transcription factor 7, T cell specific | 4.863 | 1.473 | 0.303 |
| *Tcf7l1* | transcription factor 7 like 1 (T cell specific, HMG box) | 4.045 | 2.980 | 0.737 |
| *Tle1* | transducin-like enhancer of split 1 | 13.775 | 11.987 | 0.870 |
| *Tle2* | transducin-like enhancer of split 2 | 2.907 | 1.885 | 0.649 |
| *Wif1* | WNT inhibitory factor 1 | 5.235 | 0.477 | 0.091 |
| *Wisp1* | WNT1 inducible signaling pathway protein 1 | 82.611 | 43.924 | 0.532 |
| *Wnt1* | wingless-type MMTV integration site family, member 1 | 0.011 | 0.000 | 0.000 |
| *Wnt10a* | wingless-type MMTV integration site family, member 10A | 0.000 | 0.000 | N/A |
| *Wnt11* | wingless-type MMTV integration site family, member 11 | 0.686 | 0.222 | 0.323 |
| *Wnt16* | wingless-type MMTV integration site family, member 16 | 1.631 | 0.640 | 0.392 |
| *Wnt2* | wingless-type MMTV integration site family, member 2 | 0.011 | 0.021 | 1.896 |
| *Wnt2b* | wingless-type MMTV integration site family, member 2B | 0.448 | 0.448 | 1.002 |
| *Wnt3* | wingless-type MMTV integration site family, member 3 | 0.000 | 0.017 | N/A |
| *Wnt3a* | wingless-type MMTV integration site family, member 3A | 0.000 | 0.000 | N/A |
| *Wnt4* | wingless-type MMTV integration site family, member 4 | 2.045 | 0.508 | 0.248 |
| *Wnt5a* | wingless-type MMTV integration site family, member 5A | 11.272 | 8.793 | 0.780 |
| *Wnt5b* | wingless-type MMTV integration site family, member 5B | 0.569 | 0.426 | 0.749 |
| *Wnt6* | wingless-type MMTV integration site family, member 6 | 0.011 | 0.000 | 0.000 |
| *Wnt7a* | wingless-type MMTV integration site family, member 7A | 0.000 | 0.000 | N/A |
| *Wnt7b* | wingless-type MMTV integration site family, member 7B | 0.549 | 0.657 | 1.195 |
| *Wnt8a* | wingless-type MMTV integration site family, member 8A | 0.029 | 0.000 | 0.000 |
| *Wnt9a* | wingless-type MMTV integration site family, member 9A | 1.073 | 3.156 | 2.941 |

Supplementary Table S3. Primer sequence.

| Target gene | Primer sequence |
| --- | --- |
| mouse *Gapdh* | AGG TCG GTG TGA ACG GAT TTG (Forward 5'-3') |
|  | TGT AGA CCA TGT AGT TGA GGT CA (Reverse 5'-3') |
| mouse *Zbp1* | GGG TCC CAG CTG ATG TTT CT (Forward 5'-3') |
|  | TGA AGC AAG CTG ACT TCC CT (Reverse 5'-3') |
| mouse *Runx2* | TCC ACA AGG ACA GAG TCA GAT TAC AG (Forward 5'-3') |
|  | CAG AAG TCA GAG GTG GCA GTG TCA TC (Reverse 5'-3') |
| mouse *Sp7* | ATG GCG TCC TCT CTG CTT G (Forward 5'-3') |
|  | TGA AAG GTC AGC GTA TGG CTT (Reverse 5'-3') |
| mouse *Ibsp* | ATG GAG ACG GCG ATA GTT CC (Forward 5'-3') |
|  | CTA GCT GTT ACA CCC GAG AGT (Reverse 5'-3') |
| mouse *Bglap* | CTG ACC TCA CAG ATG CCA AGC (Forward 5'-3') |
|  | TGG TCT GAT AGC TCG TCA CAA G (Reverse 5'-3') |
| mouse *CD36* | CCT GCA AAT GTC AGA GGA AA (Forward 5'-3') |
|  | GCG ACA TGA TTA ATG GCA CA (Reverse 5'-3') |
| mouse *Cebpa* | GTC ACT GGT CAA CTC CAG CA (Forward 5'-3') |
|  | TGG ACA AGA ACA GCA ACG AG (Reverse 5'-3') |
| mouse *Lpl* | GCG TAG CAG GAA GTC TGA CCA A (Forward 5'-3') |
|  | AGC GTC ATC AGG AGA AAG GCG A (Reverse 5'-3') |
| mouse *Pparg* | TGC TGT TAT GGG TGA AAC TCT G (Forward 5'-3') |
|  | CTG TGT CAA CCA TGG TAA TTT CTT T (Reverse 5'-3') |
| mouse *Axin2* | AAC CTA TGC CCG TTT CCT CTA (Forward 5'-3') |
|  | GAG TGT AAA GAC TTG GTC CAC C (Reverse 5'-3') |
| mouse *Ccnd1* | GCG TAC CCT GAC ACC AAT CTC (Forward 5'-3') |
|  | ACT TGA AGT AAG ATA CGG AGG GC (Reverse 5'-3') |
| human *GAPDH* | GGA GCG AGA TCC CTC CAA AAT (Forward 5'-3') |
|  | GGC TGT TGT CAT ACT TCT CAT GG (Reverse 5'-3') |
| human *ZBP1* | GCA AAC TCC GAA GCC ATC CAG A (Forward 5'-3') |
|  | CCA AGT TGA GGA ATC ACC TGG TG (Reverse 5'-3') |
| human *RUNX2* | TGG TTA CTG TCA TGG CGG GTA (Forward 5'-3') |
|  | TCT CAG ATC GTT GAA CCT TGC TA (Reverse 5'-3') |
| human *SP7* | TTC TGC GGC AAG AGG TTC ACT C (Forward 5'-3') |
|  | GTG TTT GCT CAG GTG GTC GCT T (Reverse 5'-3') |
| human *COL1A1* | GAT TCC CTG GAC CTA AAG GTG C (Forward 5'-3') |
|  | AGC CTC TCC ATC TTT GCC AGC A (Reverse 5'-3') |
| human *SPP1* | CGA GGT GAT AGT GTG GTT TAT GG (Forward 5'-3') |
|  | GCA CCA TTC AAC TCC TCG CTT TC (Reverse 5'-3') |
| human *CD36* | CAG GTC AAC CTA TTG GTC AAG CC (Forward 5'-3') |
|  | GCC TTC TCA TCA CCA ATG GTC C (Reverse 5'-3') |
| human *CEBPA* | AGG AGG ATG AAG CCA AGC AGC T (Forward 5'-3') |
|  | AGT GCG CGA TCT GGA ACT GCA G (Reverse 5'-3') |
| human *LPL* | CTG CTG GCA TTG CAG GAA GTC T (Forward 5'-3') |
|  | CAT CAG GAG AAA GAC GAC TCG G (Reverse 5'-3') |
| human *PPARG* | AGC CTG CGA AAG CCT TTT GGT G (Forward 5'-3') |
|  | GGC TTC ACA TTC AGC AAA CCT GG (Reverse 5'-3') |
| Chip *Runx2* prompter | TGG TAG GCA GTC CCA CTT TAC TTT (Forward 5'-3') |
|  | GGC GAA TGA AGC ATT CAC AC AA (Reverse 5'-3') |
| Chip *Runx2* upstream | GAC TCC TTT CAG GCA CCA TC (Forward 5'-3') |
|  | TCC TGG AGA ACA CAG AAC CC (Reverse 5'-3') |
| Chip *Sp7* prompter | CCA TGT GGG TAG CAG AGA GT (Forward 5'-3') |
|  | GGG ACG ACA CTC ACA CAG AA (Reverse 5'-3') |
| Chip *Sp7* upstream | GAA CAG GAA TCA GGG AGC CT (Forward 5'-3') |
|  | TCC TGG AGA ACA CAG AAC CC (Reverse 5'-3') |
| Chip *Zbp1* WRE1 | TTC GAT TTC GAC ACC CTT TC (Forward 5'-3') |
|  | TGA ATC CAC AGG CAA GAA TG (Reverse 5'-3') |
| Chip *Zbp1* WRE2-3 | ATT GAT GAG GTC ACC AAG GC (Forward 5'-3') |
|  | TAG GGC AGG AGA AGA GCT TG (Reverse 5'-3') |
| Chip *Zbp1* downstream | AAA TTG CCC AAC TCC AGC TG (Forward 5'-3') |
|  | AGA TGT ACT GAG CTC CGT GG (Reverse 5'-3') |

**Supplementary Fig. S1**, related to Fig. 1

Depletion of ZBP1 slightly inhibited proliferation of mBMSCs. **a** MTT cell-proliferation assay of ZBP1-depeleted mBMSCs and control cells at day 0, 1, 4, 7, 10. **b** RT-qPCR analysis and western blot analysis of ZBP1 expression in mBMSCs infected with shZBP1 lentiviruses and scrambled shRNA control.

**Supplementary Fig. S2**, related to Fig. 6

Quantification of Fig. 6h and expression of *Runx2* and *Sp7* upon ZBP1 depletion in mBMSCs. **a** Quantification of three representative western blots of β-catenin in NE and CE of ZBP1-depleted mBMSCs and control cells. **b** Fragments per kilobase of transcript per million (FPKM) mapped reads (FPKM) value of *Runx2* and *Sp7* in ZBP1-depleted mBMSCs and control cells. **c** RT-qPCR analysis of *Runx2* and *Sp7* expression in ZBP1-depleted mBMSCs and control cells.
