# Supplementary figures and images for: ZBP1 (DAI/DLM-1) promotes osteogenic differentiation while inhibiting adipogenic differentiation in mesenchymal stem cells through a positive feedback loop of Wnt/β-catenin signaling
Source: Bone Res. 2020 Mar 5;8:12. doi: 10.1038/s41413-020-0085-4 (PMC7058036; doi:10.1038/s41413-020-0085-4)

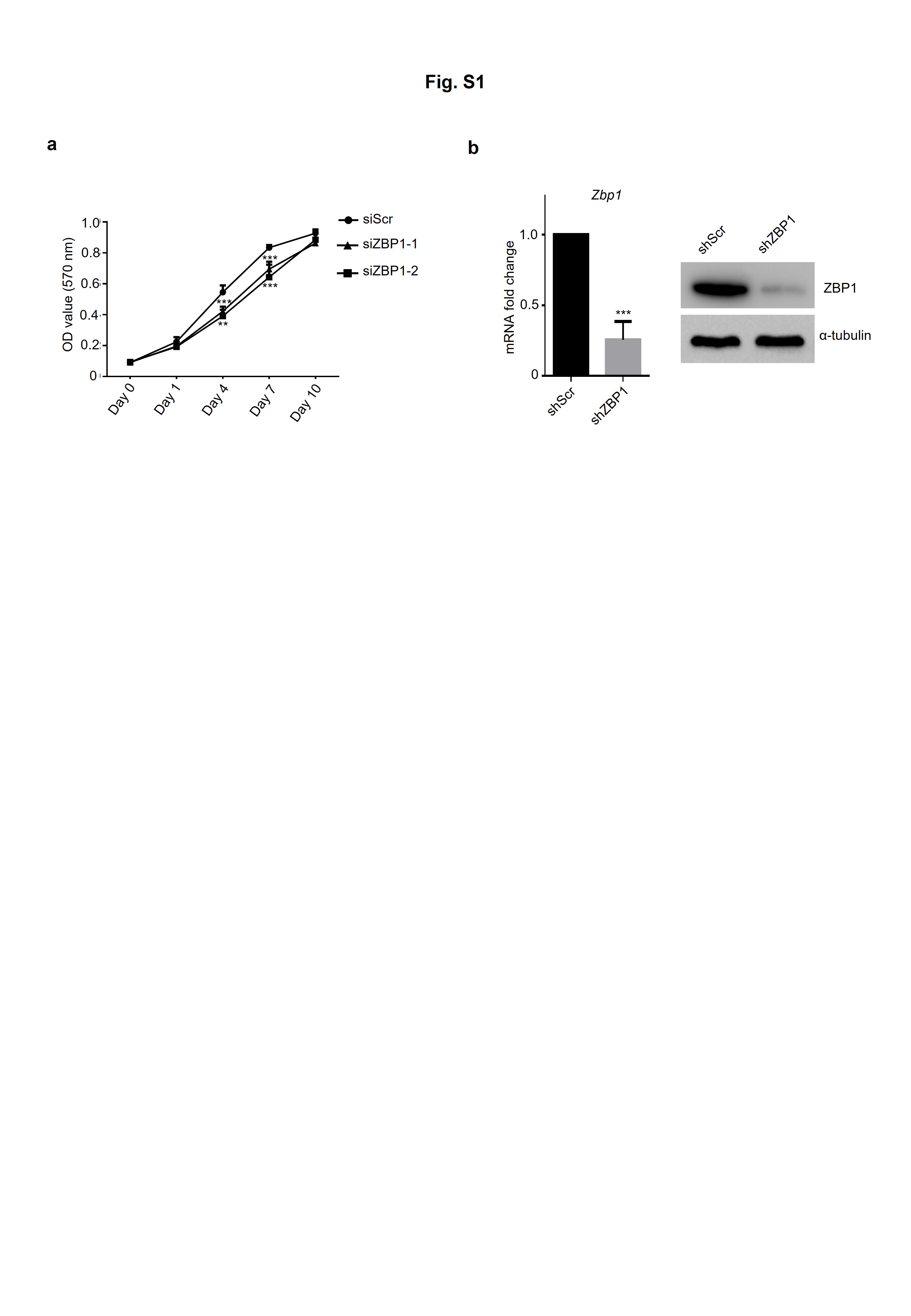

Supplement: Supplementary file 2 — Figure S1 [file 41413_2020_85_MOESM2_ESM.jpg]

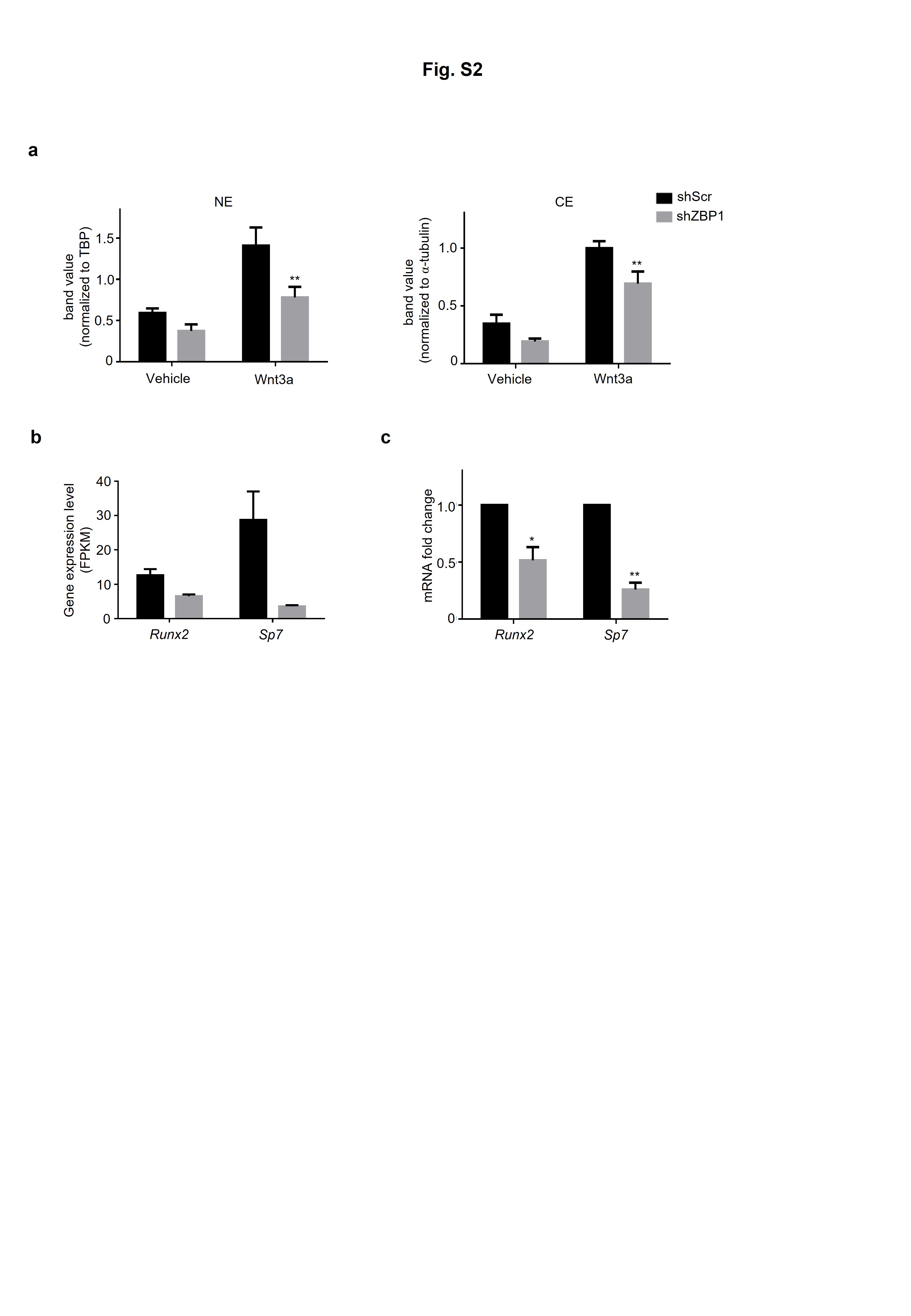

Supplement: Supplementary file 3 — Figure S2 [file 41413_2020_85_MOESM3_ESM.jpg]
